# Catalyst-based biomolecular logic gates

**DOI:** 10.3390/catal12070712

**Published:** 2022-06-29

**Authors:** Dennis S. Winston, David D. Boehr

**Affiliations:** 1 Department of Chemistry, The Pennsylvania State University, University Park, PA, USA, 16802

**Keywords:** logic gate, allostery, enzyme computing, DNA computing, protein engineering, metabolic engineering, enzyme cascade

## Abstract

Regulatory processes in biology can be re-conceptualized in terms of logic gates, analogous to those in computer science. Frequently, biological systems need to respond to multiple, sometimes conflicting, inputs to provide the correct output. The language of logic gates can then be used to model complex signal transduction and metabolic processes. Advances in synthetic biology in turn can be used to construct new logic gates, which find a variety of biotechnology applications including in the production of high value chemicals, biosensing and drug delivery. In this review, we focus on advances in the construction of logic gates that take advantage of biological catalysts, including both protein-based and nucleic acid-based enzymes. These catalyst-based biomolecular logic gates can read a variety of molecular inputs and provide chemical, optical and electrical outputs, allowing them to interface with other types of biomolecular logic gates or even extend to inorganic systems. Continued advances in molecular modeling and engineering will facilitate the construction of new logic gates, further expanding the utility of biomolecular computing.

## 1. Introduction

Organisms have evolved a variety of mechanisms to respond, communicate and adapt to their environments. For example, fundamental cellular processes including transcription and translation are highly regulated. Protein function, especially enzyme function, is often modulated by the concentrations of metabolites and signaling molecules. The activity of many enzymes depends not just on the concentration of substrate, but on other metabolites that bind to allosteric sites, allowing for more complex regulation. The interconnected web of the reactions and regulation involved in metabolism can be conceptualized as a circuit of logic gates, analogous to a computer, where each individual reaction is a logic gate that produces output molecules in response to input molecules. In the case of an enzyme allosterically regulated by metabolites, the “decision” to produce more or less of a given metabolite depends on the concentration of the metabolites that control the activity of the enzyme. As such, biological regulation can be re-conceptualized in terms of logic gates, which then has a wide variety of biotechnology applications, including in synthetic biology for optimized production of high value chemicals (e.g. [1,2]), for engineering of biosensors that sense multiple analytes (e.g. [3–5]), and for the programmed delivery of therapeutics based on biomarkers (e.g. [6,7]). In these applications, synthetic biological logic gates are often designed to interface with the existing biochemical logic gates in the cell. Here we provide some historical background for understanding biomolecular logic gates, outline recent work conceptualizing allosteric enzymes as logic gates, describe how enzymes (and their associated chemical reactions) can be engineered to behave as logic gates in response to biological inputs and finally expand the discussion to nucleic acid-based logic gates, where allosterically regulated DNA-based enzymes (DNAzymes) can play important roles.

### 1.1 Fundamentals of logic gates

Logic gates are the basic building blocks of computers. In electronic Boolean logic gates, a low voltage/current is 0 and a high voltage/current is 1. This concept can be expanded to chemical reactions, where a low signal is 0 and a high signal is 1. Although the inputs and outputs of a molecular logic gate are not truly binary, molecular circuits have been designed to behave closer to binary logic gates with a sigmoidal response to inputs [8–12] though such behavior is difficult to apply generally. In living cells, biomolecules sense and transmit signals, behaving as complex circuits made of logic gates [13,14]. It has been shown theoretically that a series of chemical reactions occurring in solution without compartmentalization can be used to create logic circuits of arbitrary complexity [15] enabling mathematical operations to be carried out [16], although for multi-component systems noise reduction techniques may be necessary [17,18]. While our focus in this review is on idealized binary biomolecular logic gates, logic circuits in synthetic biology can be more generally understood using the theoretical framework of analog circuits to take into account the noise and leakiness that occur in real molecular systems [19,20].

A logic gate returns an output based on one or more inputs (Table 1). The simplest logic gate is the YES (or IDentity) gate, where the output is the same as the input. The YES gate is trivial to implement on its own but can be used as part of more complex logic operations. The NOT gate is the inverse of the YES gate, where the output is the opposite of the input. The YES and NOT gates can be implemented in biomolecular systems when a signal based on the product of a chemical reaction depends on the presence or absence of a reactant. The AND gate results in an output of 1 only if both inputs are 1. The OR gate results in an output of 1 if one or both inputs are 1. The AND and OR gates are the most commonly used biomolecular logic gates because of their relative simplicity. The NAND and NOR gates are the same as the AND and OR gates but with the output inverted. The XOR gate has an output of 1 only if one of the inputs, but not both, is 1. The NXOR gate is an XOR gate with inverted output. The NAND and NOR logic gates, while difficult to implement using biomolecules, are functionally complete operations, meaning they can be combined to produce any other logic operation. The inhibit gate (INH) describes a system in which the output is 1 when input A is 1, but is 0 when input B is 1. For example, if input A is the substrate for an enzyme and the output is the reaction product, input B is an inhibitor of the enzyme. The imply gate (IMP) is an INH gate with the output reversed. A summary of the fundamental logic gates and biomolecular examples is provided in [21].

### 1.2 Early history of biomolecular logic gates

A formal conception of chemical reactions as logic gates was published in 1961 [22]. Molecular logic gates have been developed for small molecules, but we will not focus on that work here and refer readers to other reviews in this area (e.g. [23–27]). In a biological context, the concept of molecular logic gates was initially applied to transcriptional regulation of enzyme activity [28,29]. In the 1980s, Okamoto and co-workers [30,31] applied the previously developed mathematical framework [32] directly to enzyme-catalyzed reactions. A decade later, the switching between metabolic pathways involving glucose was conceptualized by logic gates, and the dependence of the sharpness of transition between logical states on enzyme kinetic parameters was analyzed [33]. In one of the first experimental demonstrations of an enzyme-based logic gate, an AND gate was created by conformational switching of enzyme-conjugated azobenzene derivatives by light and oxidation/reduction of an inhibitor to control α-chymotrypsin activity [27,34]. In another experimental example, an XOR gate was realized via the Ca^2+^ and Mg^2+^ dependence of malate dehydrogenase activity [35]. Willner and colleagues [36,37] developed a half-adder and half-subtractor from parallel XOR and AND gates using horseradish peroxidase, glucose dehydrogenase, glucose oxidase, and catalase in the presence of reduced (NADH) and oxidized (NAD^+^) forms of nicotinamide adenine dinucleotide. The first experimental demonstration of consecutive enzyme-based logic gates was in 2006, where a system of acetylcholine esterase, choline oxidase, microperoxidase-11, and glucose dehydrogenase was used to create logic circuits composed of concatenated AND, OR, and XOR gates [38]. In most examples of enzyme-based logic gates, output signals have been observed by spectrophotometry, although other forms of output are possible, including detecting changes in pH as an electrical signal [39,40] and interfacing to DNA computers [41]. Other methods for sensing outputs and additional examples are provided in [42]. We note that there are many strategies for designing biomolecular logic gates and circuits, including modulation of protein-protein interactions or dimerization [43,44] and regulation of transcription [45,46] including distributed computing across populations of cells [47,48], however in this review, we will focus primarily on catalyst-based logic gates that feature protein enzymes, nucleic acid enzymes, and catalytic DNA strands.

## 2. Protein-based logic gates

### 2.1 Allosteric enzymes as logic gates

**Figure 1.**
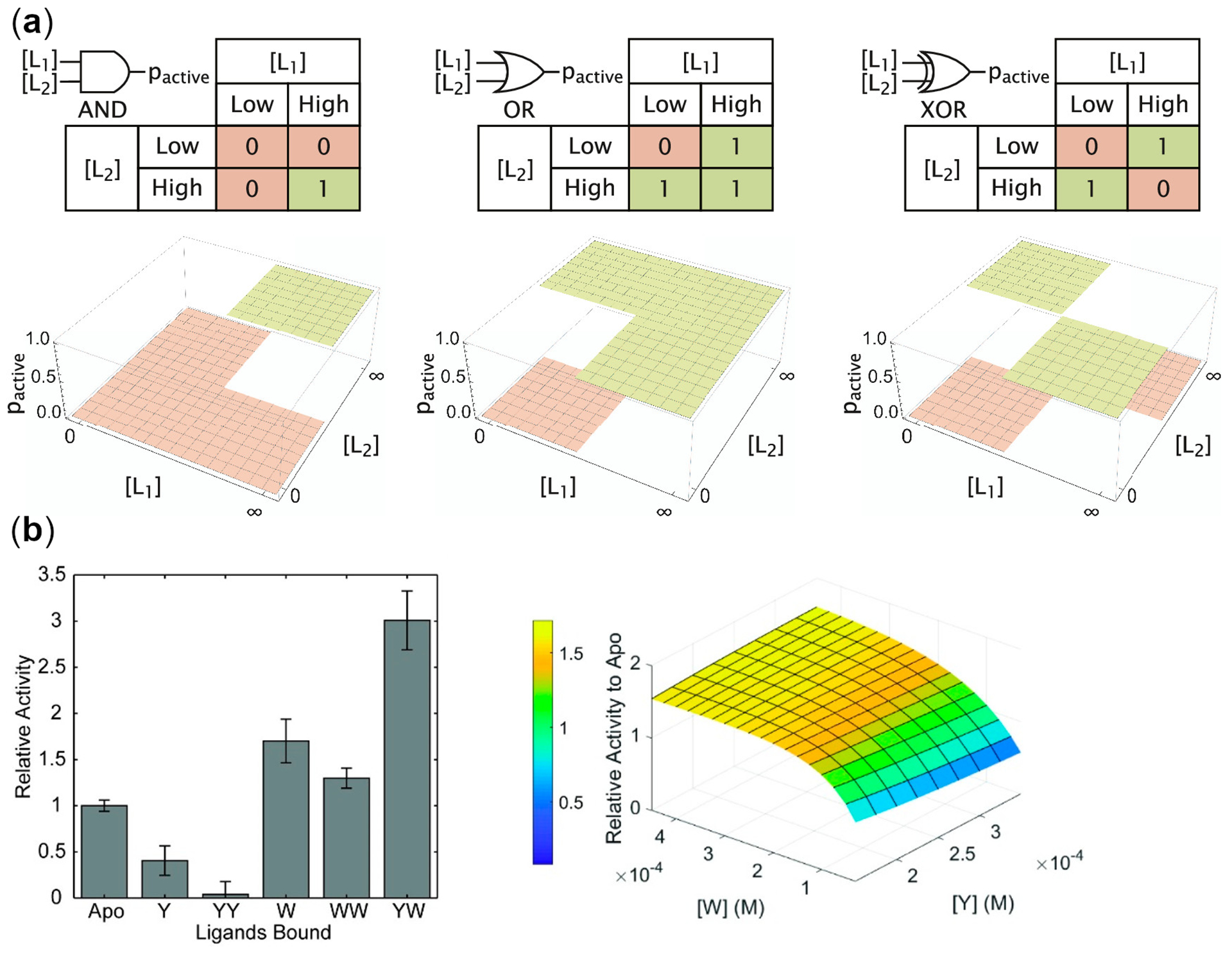
Allosteric enzymes as logic-gates. **A.** Binding of different allosteric modulators (L_1_ and L_2_) can result in different functional output (i.e. 0 for no or low enzyme activity; 1 for high enzyme activity). This panel is Reprinted with permission from Galstyan, V., Funk, L., Einav, T. and Phillips, R. 2019. Combinatorial control through allostery. J. Phys. Chem. B., 123, 2792–2800. Copyright 2019 American Chemical Society [56]. **B.** The functional landscape of *Saccharomyces cerevisiae* chorismate mutase (ScCM) is dependent on the concentration of the allosteric modulators tryptophan (W) and tyrosine (Y). ScCM is a homodimer in which the allosteric site binds W or Y (i.e. binding is mutually exclusive at each site). When ScCM binds Y at one site and W at the other site, ScCM displays the highest activity, which could be conceptualized as an AND gate. However, binding of Y alone leads to allosteric inhibition, providing complexity beyond the binary logic gates. This panel is Reprinted with permission from Gorman, S.D. and Boehr, D.D. 2019. Energy and enzyme activity landscapes of yeast chorismate mutase at cellular concentrations of allosteric effectors. Biochemistry, 58, 4058–4069. Copyright 2019 American Chemical Society [49].

In this review, we focus on recent work using biological catalysts to create biomolecular logic gates. Our own work has focused on understanding allostery and conformational dynamics of enzymes (e.g. [49–52]), which has implications in the development of biomolecular logic gates. Allosteric regulation can allow enzymes to behave as biomolecular logic gates [33]. For example, pyruvate kinase from *Mycobacterium tuberculosis* that uses adenosine monophosphate (AMP) and glucose-6-phosphate (G6P) as synergistic allosteric activators has been conceptualized as acting as an OR gate to regulate energy and glucose metabolism [53]. Binding of AMP and G6P induce similar allosteric pathways that likely help to regulate the active site. The sigmoidal response of enzymes that are allosterically activated by substrate binding can be useful for reducing noise [54]. Allosteric enzymes that follow the Monod-Wyman-Changeux (MWC) model of allostery, in which concerted conformational transitions occur across enzyme subunits, have been analyzed as logic gates and single-output molecules with two inputs have been found to be capable of producing YES, NOT, AND, OR, NOR, or NAND gates depending on the system [55,56] (Figure 1).

### 2.2 Engineered protein logic gates

**Figure 2.**
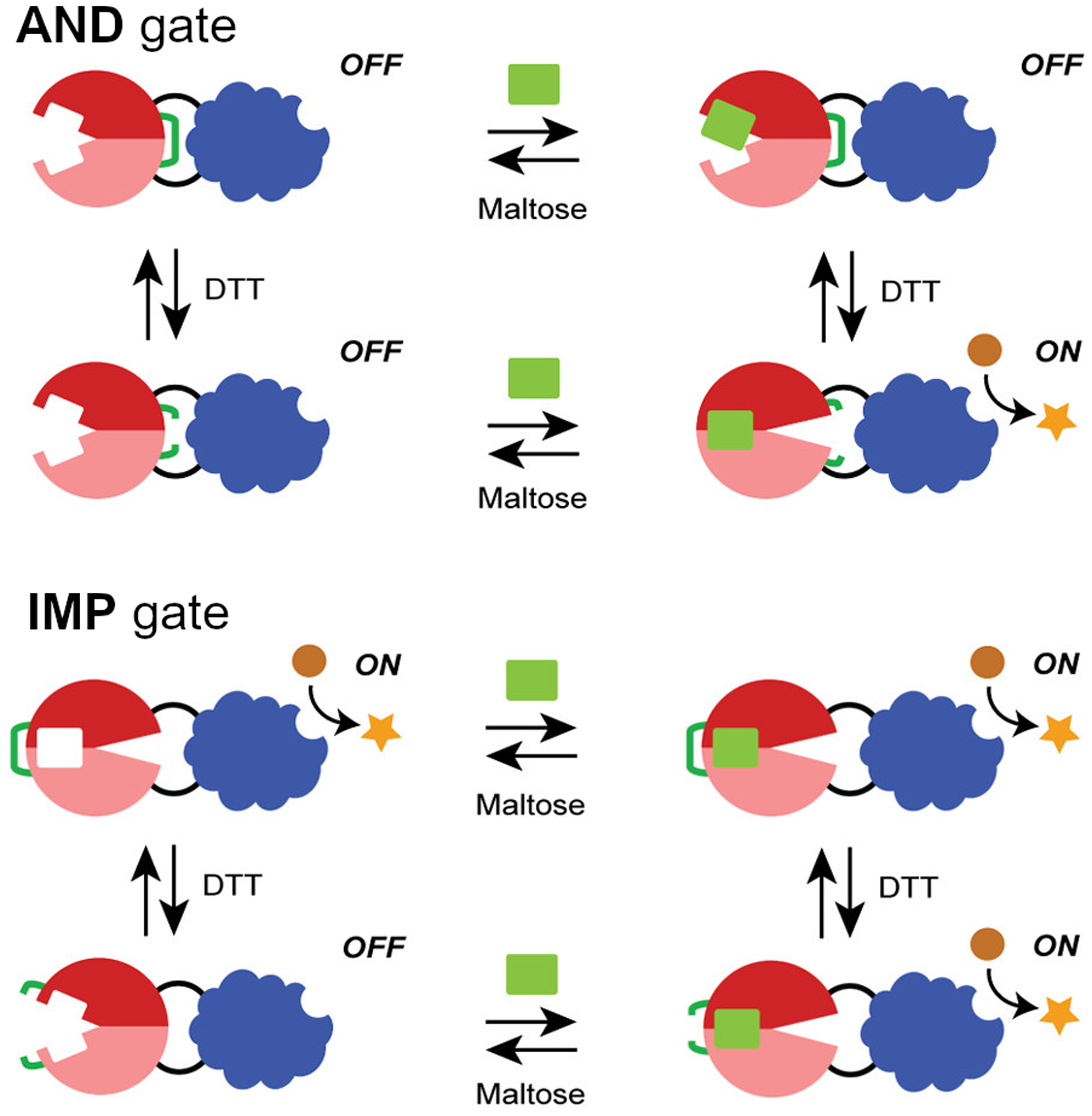
Redesign of logic gates using engineered disulfide bridges. The activity of BLA (blue) is dependent on the ability of MBP (red) to bind to maltose. In the AND gate design, a disulfide bond (green) holds the MBP domain in an open conformation, preventing the conformational change in MBP necessary to activate BLA. Addition of the DTT reducing agent breaks the disulfide bond to allow the MBP conformational change and BLA activation to occur. In the IMP gate design, the engineered disulfide bond now holds MBP in the closed conformation, such that BLA is always activated unless DTT is present and maltose is absent. This figure is adapted and Reprinted with permission from Choi, J.H. and Ostermeier, M. 2015. Rational design of a fusion protein to exhibit disulfide-mediated logic gate behavior. ACS Synthetic Biology, 4, 400–406. Copyright 2015 American Chemical Society.

Chimeric proteins have been created to act as logic gates. Ostermeier’s group has designed fusions of β-lactamase (BLA) and maltose binding protein (MBP) that relies on a large conformational change induced by binding of maltodextrins to activate β-lactamase [57–59]. The sequence for BLA was randomly divided and these two parts were inserted around a MBP sequence to mimic genetic recombination and proteins with reversible maltose-dependent BLA activity were identified [57]. This MBP-BLA fusion protein can be conceptualized as a variety of logic gates depending on the input. It functions as a YES gate for maltose, as BLA activity occurs in the presence of maltose but not in its absence. It is an OR gate for maltose and maltotriose, as either maltodextrin activates BLA activity. It is an IMP gate for maltose and β-cyclodextrin because β-cyclodextrin binding results in only a small conformational change that does not confer BLA activity [60] (Figure 2). They subsequently used directed evolution to select for sucrose-dependent BLA activity, creating an OR gate with sucrose and maltose as inputs [58]. They then engineered disulfide bonds into the MBP-BLA fusion to lock it in either the active/closed or inactive/open state in the absence of reducing agent. With disulfide bonds to lock MBP-BLA in the closed/active state, the protein behaves as an IMP gate with maltose and reducing agent as inputs; that is, in the absence of reducing agent this variant is active, but in the presence of reducing agent maltose is required for activity. With disulfide bonds to lock the protein in the open/inactive state, the protein acts as an AND gate for maltose and reducing agent i.e. protein only has a chance to be in the active state in the presence of reducing agent and needs maltose to be activated. With disulfide bonds in the hinge region and mutations that destabilize the open/inactive state, the BLA activity is regulated by reducing agent but not maltose, converting it from a YES gate for maltose to a YES gate for reducing agent. If oxidizing agent is used as an input instead of reducing agent, the IMP gate becomes an OR gate and the AND gate becomes an INH gate [60]. In general, using reducing agent as an input for enzymatic logic gates may be useful for therapeutics by activating or inactivating enzymes upon entry to the cytosol.

**Figure 3.**
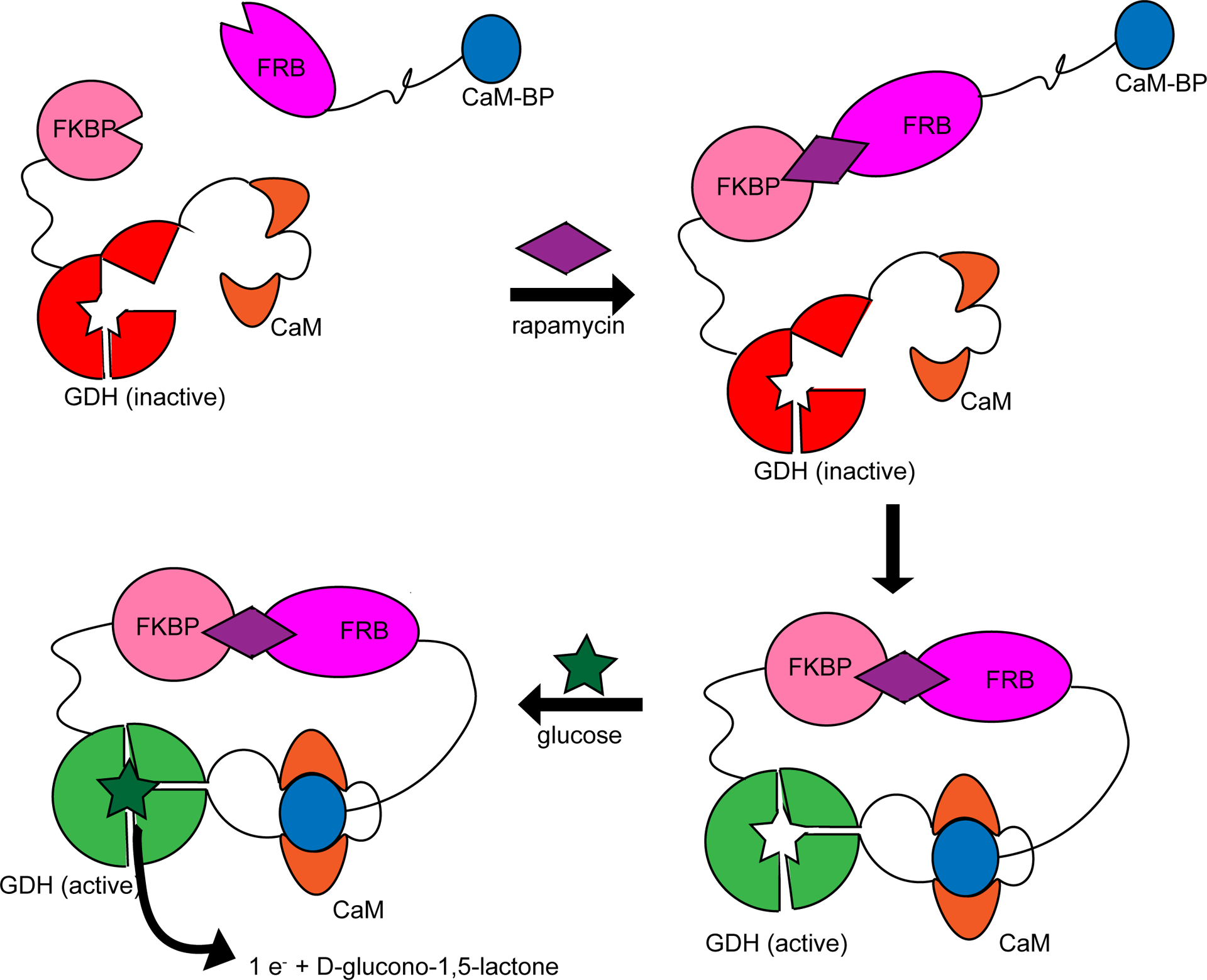
Calmodulin-glucose dehydrogenase (CaM-GDH) fusion protein that acts as an AND gate with rapamycin and glucose as inputs. Upon rapamycin binding, calmodulin binds calmodulin-binding peptide (CaM-BP). Binding of CaM-BP results in a conformational change in GDH, activating the enzyme and allowing it to catalyze the oxidation of glucose and produce an electrical signal as output. The electrical signal is produced only in the presence of both rapamycin and glucose.

Another clever system that has been engineered to behave as a logic gate is pyrroloquinoline quinone-dependent glucose dehydrogenase (PQQ-GDH) fusion proteins, in which PQQ-GDH is fused to a reporter domain that binds a given ligand. GDH activity is allosterically regulated by ligand binding to the reporter domain. An advantage of using PQQ-GDH is that PQQ-GDH catalyzes a redox reaction, allowing the signal to be easily incorporated into an electronic circuit. Guo and colleagues [61] converted PQQ-GDH into an allosteric enzyme regulated by peptide binding by inserting calmodulin (CaM) at a loop by the glucose binding site, reducing GDH activity in the absence of peptide (Figure 3). To prevent activation at low peptide concentrations, they engineered calmodulin binding peptide (CaM-BP) to have lower affinity for CaM and fused it with the FRB protein. They then fused the GDH-CaM to FK506 binding protein (FKBP), which binds FRB in the presence of rapamycin. By increasing rapamycin concentration, FKBP binds FRB which then causes the local concentration of CaM-BP to increase, activating GDH. In this way, they were able to regulate the activity of GDH by adjusting the rapamycin concentration. They also showed that a variety of both ligands and reporters can be used. For example, they fused CaM-BP with different antibody domains to detect the proteins α-amylase, thrombin-activatable fibrinolysis inhibitor, clostridium TcdaA toxin, and interleukin-23. To demonstrate that different reporters can be used, instead of GDH activity they also used dihydrofolate reductase, green fluorescent protein (EGFP), and NanoLuciferase as reporters. Using the synthetic peptide receptor Clamp instead of CaM resulted in ligand binding turning off enzyme activity instead of turning it on. A further layer of regulation can be added to the CaM fusion system by adding a calmodulin domain to the CaM-BP to sequester CaM-BP that can be removed by protease cleavage to activate calmodulin [62]. The CaM fusion systems described here can be considered as four-input logic gates with analyte, substrate, cofactor, Ca^2+^ as inputs [63], although for biosensing applications the substrate, cofactor, and Ca^2+^ are always present so it effectively behaves as a YES gate for the analyte.

Subsequent work by the Katz group [64] used the electrochemical signal generated by the PQQ-GDH-Clamp enzyme’s reduction of glucose to release molecules into solution. They set up an IMP logic gate using Clamp’s peptide and a PQQ-GDH without Clamp as input. In this system, PQQ-GDH-Clamp, glucose substrate and cofactor were always present in solution. Because GDH-Clamp is inhibited by peptide, but the GDH without Clamp can still reduce glucose in the presence of substrate, the reaction occurred for all cases except when both peptide and PQQ-GDH were absent. A current was produced by either conjugating the two PQQ-GDH enzymes to carbon nanotube buckypaper electrodes via an ester linker that both reacts with protein lysine residues and binds to the nanotubes or mediating electron transfer from solution via 2,6-dichlorophenolindophenol (DCPIP) and phenazine methosulfate (PMS) [65]. This current was then used to drive the release of a molecule into the solution. The current generated by the reduction of glucose was coupled with the reduction of water catalyzed by bilirubin oxidase, which resulted in a local pH increase leading to hydrolysis of an ester. The cleavage of the ester released the molecule into solution. While they released a fluorescent dye, in principle any molecule that can be conjugated to the carbon nanotube could be released. Combined with the customizable GDH-CaM fusion proteins, the ability to release molecules via an enzyme-catalyzed reduction reaction can be used to release molecules or detect electric current in response to a wide variety of analytes.

Dokholyan’s group constructed an OR gate in live cells by engineering focal adhesion kinase (FAK) to respond to chemical and optical inputs [66]. FAK is a kinase involved in cytoskeletal regulation and is allosterically regulated by the binding of regulatory proteins in its FERM domain. They inserted a previously constructed regulatory domain that allows for control of FAK activity via binding of rapamycin [67], with the enzyme in an open active conformation in the presence of rapamycin. To regulate the enzyme by optical irradiation, they inserted a Light Oxygen Voltage 2 (LOV2) domain, which undergoes a conformational change in response to blue light, into a loop in the regulatory FERM domain determined to allosterically couple to the active site by computational analysis [68]. Upon irradiation, the kinase switches to an inactive closed conformation. First, constructs with the rapamycin-binding domain and LOV2 domain inserted separately were tested in HeLa cells and found to undergo regulation as expected. Because FAK activity results in the formation of large focal adhesions, the size of focal adhesions was used as the output for the logic gate. Then light- and dark-stabilized variants of FAK with both allosteric regulatory domains inserted were used to test the logic gate behavior (due to phototoxicity, it was not possible to apply different combinations of rapamycin and light inputs at the same time.). In the dark, FAK was always active independent of rapamycin concentration. In the light, FAK was activated by addition of rapamycin. Therefore when the rapamycin and absence of blue light are considered inputs and FAK activity is the output, the system behaves as an OR gate.

Ostermeier’s group [69] used a pH-sensitive variant of the membrane protein listeriolysin O, which forms pores at low pH, to selectively release molecules from liposomes. By conjugating a designed ankyrin repeat protein (DARPin)-based inhibitor to the membrane, they controlled pore formation by releasing the inhibitor in response to either reducing agent or protease cleavage. With pH, protease, and reducing agent as inputs, the system can be conceptualized as an AND-OR gate, where low pH in combination with either protease or reducing agent results in pore formation. Under these conditions, the fluorescent probe calcein was released from the vesicles. It is noted that the enzyme serves as an input here, rather than as a gate itself.

### 2.3 Biosensors based on enzymatic logic gates

**Figure 4.**
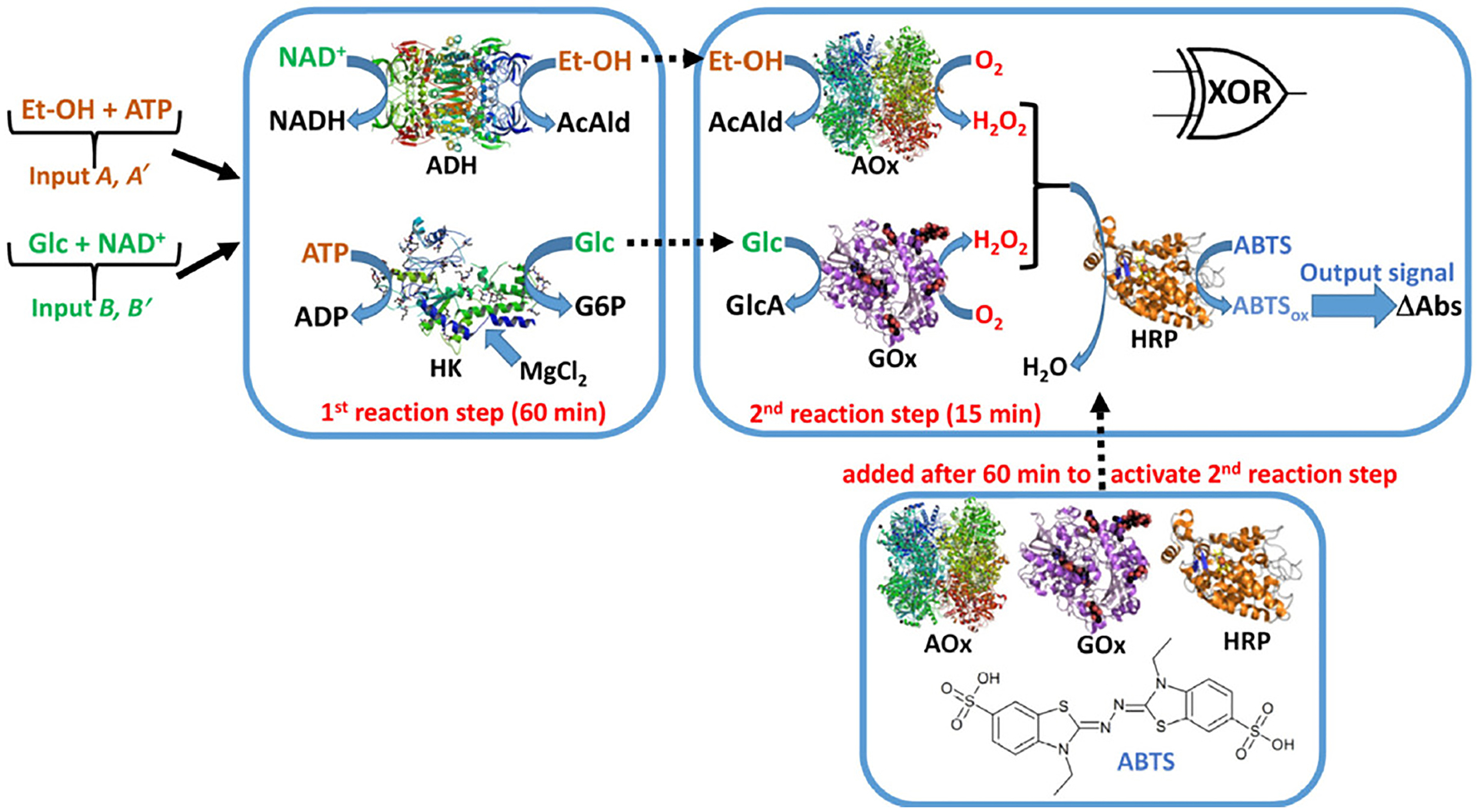
An enzyme cascade to model a XOR gate. When both inputs are provided (EtOH/ATP and Glc/NAD^+^), the required substrate and cofactor are available for the ADH and HK enzymes resulting in the removal of the required substrate for the second series of enzymes, AOx and GOx and no production of H_2_O_2_. However, if only one of the inputs is provided, the EtOH or Glc substrates for AOx or GOx, respectively, will be available for H_2_O_2_ production. The figure was adapted with permission from [75].

The chemical reactions catalyzed by a series of different enzymes can also be constructed to act as logic gates (e.g. see Figure 4). For example, Katz and Privman [70] used the controlled release of hydrogen peroxide (H_2_O_2_) in response to a logic gate made of a four-level enzyme cascade to release molecules from an Fe^3+^ cross-linked alginate hydrogel. Prior to this work, only two enzymatic reactions were able to be carried out in sequence with the enzymes immobilized on the hydrogel [71]. Their system consisted of four enzymes deposited on the interface of the alginate hydrogel film and the solution, which could be conceptualized as a series of interconnected AND gates where the inputs are the reactants in different steps of the cascade that are not products of a previous step. The first enzyme in the cascade was amyloglucosidase (AMG), which catalyzes the hydrolysis of maltose to two glucose molecules. The next enzyme was glucose dehydrogenase (GDH), which oxidizes glucose and reduces NAD^+^ to NADH. The next enzyme was lactate dehydrogenase (LDH), which catalyzes the reduction of pyruvate into lactate with NADH as a cofactor. The final enzyme was lactate oxidase (LOx), which catalyzes the oxidation of lactate by O_2_ to produce pyruvate and H_2_O_2_. The hydrogen peroxide then diffuses into the hydrogel and destroys it via a free radical mechanism, releasing the reporter molecule. The reporter molecule used was a fluorescent DNA, which in principle could be used as an input to DNA logic gates (e.g. see below) or could be a small molecule drug or input for downstream enzymatic reactions.

Katz’s group also created a reversible CNOT logic gate using two enzymatic reactions resulting in pH changes in opposite directions [72]. First, a XOR gate was created using two enzymes, urease and esterase, conjugated to the surface of a sensitive pH detector. Urease catalyzes the hydrolysis of urea, resulting in production of ammonia and an increase in pH, while esterase catalyzes the hydrolysis of ethyl butyrate, resulting production of butyric acid and in a decrease in pH. In the presence of either urea or ethyl butyrate, the pH at the surface of the detector changes drastically and a large absolute value of pH change is considered an output of 1. However, when urea and ethyl butyrate are present together, a weak acid and weak base are produced simultaneously, resulting in only a small pH change and an output of 0. In this way, an XOR gate was created. A similar XOR gate was also constructed using urease and penicillinase immobilized on a pH-sensing device, with urea and penicillin as inputs [73]. This XOR gate was run in parallel with a separate detector with only esterase conjugated to it. When the esterase-only detector senses a large pH change, ethyl butyrate is present, resulting in a YES gate. When considered in parallel with the XOR gate, the combined logic circuit has two inputs and two outputs, with each combination of outputs corresponding to a unique combination of inputs, hence the logic gate is reversible. They also constructed a XNOR gate, where the output is 1 only when both inputs have the same value, by using pH as an input [74]. They used glucose oxidation by PQQ-GDH as the gate, with the enzymatic activity depending on the pH. After mapping the enzyme activity as a function of pH and pH as a function of acetic acid and ammonia concentration, they were able to define thresholds to convert the enzyme activity into a binary output. By controlling the pH via concentration of the input molecules, the XNOR gate was made. Together, logic gates that use pH as an output and input can be combined to construct multi-layered enzyme logic circuits.

Katz’s group created two other enzyme-based XOR gates. The first system consisted of two sets of enzymes, with the second set of enzymes added after the first set of reactions was given enough time to reach completion [75] (Figure 4). The first set of enzymes consisted of alcohol dehydrogenase (ADH) and hexokinase (HK). ADH consumes ethanol (EtOH) only in the presence of NAD^+^ and HK consumes glucose only in the presence of ATP. The second set of enzymes consisted of alcohol oxidase (AOx) and glucose oxidase (GOx). AOx uses EtOH to produce H_2_O_2_ and GOx uses glucose to produce H_2_O_2_. Production of H_2_O_2_ is considered the output, as it is used by horseradish peroxidase to produce a spectrophotometric signal. The system used two inputs: 1) EtOH and ATP and 2) glucose and NAD^+^. The key concept is that in the presence of both inputs, EtOH and glucose are both fully consumed by the first enzyme set so that no H_2_O_2_ is produced after addition of the second enzyme set. When only one input is added, the enzyme that would consume either EtOH or glucose in the first set does not have its cofactor and so the EtOH or glucose will be present in solution for the second enzyme set to use to produce H_2_O_2_. In the absence of both inputs, there is no glucose or EtOH so no H_2_O_2_ is produced.

In later work, Katz’ group constructed a different XOR gate to release nanoparticles from an alginate hydrogel by production of H_2_O_2_ [76]. Two enzymes, GOx and L-glutamate oxidase (GluOx), were immobilized on the hydrogel surface. The solution contained two more enzymes: PQQ-GDH and glutamate dehydrogenase (GluDH). The two inputs to the XOR gate were 1) PMS (cofactor for GDH) and glutamate (substrate for GluDH and GOx), and 2) NAD^+^ (cofactor for GluDH) and glucose (substrate for GDH and GluDH). If H_2_O_2_ is produced at the surface of the hydrogel i.e. by GOx or GluOx, the H_2_O_2_ decomposes the hydrogel to release a nanoparticle. In the absence of any input, the nanoparticle is not released from the hydrogel because no H_2_O_2_ is formed. When just NAD^+^ and glucose were present, the glucose could not be reduced by GDH without PMS, so it was oxidized by GOx, producing H_2_O_2_ and releasing the nanoparticle. When just PMS and glutamate were present, glutamate was oxidized by GluOx, again resulting in production H_2_O_2_ and nanoparticle release. When both inputs were present (all four molecules), glucose was reduced by the GDH in solution before it could be oxidized by GOx on the hydrogel surface, preventing production of H_2_O_2_. Similarly, the glutamate was reduced by GluDH before it could be oxidized by GluOx, preventing production of H_2_O_2_. This system is a notable advancement over previous work, as time-dependent addition of enzyme is not required. The nanoparticle released from this system can in principle be used for colorimetric sensing, as an input for further logic circuits, or for targeted drug delivery.

### 2.4 Protease-based logic gates

A number of biomolecular logic circuits have made use of proteases to control protein-protein interactions and transcription. As these systems have been extensively reviewed in recent articles [77–79], we will focus here on two strategies for constructing protease-based cellular logic gates that rely on protein-protein interactions. Elowitz’ group developed an elegant system of logic gates with viral proteases as inputs, called circuits of hacked orthogonal modular proteases (CHOMP) [80]. The output comes from a target protein that is activated or inactivated upon cleavage by a protease. The target protein is regulated by being linked with a protease cleavage site to a degron so that in the absence of protease the target is degraded. They created an AND gate by linking the target protein and degron by two sequential protease cleavage sites for two separate proteases. They created an OR gate by using two degrons with separate cleavage sites at the N- and C-termini of the target protein so that either protease will lead to degradation of the target. Negation was achieved by using protease cleavage to reveal the degrons instead of removing them. To achieve more complex regulation, the protease itself was split in half, constituted by two leucine zipper domains that can be cleaved by a different, secondary protease. Moving beyond binary logic gates, they created a bandpass circuit where target protein activity increased over a period of hours before returning to the baseline over a day. The bandpass circuit was constructed by using both activator and repressor proteases (cleaving one degron, revealing another) and setting a threshold for the repressor protease by inactivating it with a third protease. They applied the CHOMP system to develop a proof of concept for a programmable therapeutic device by activating caspase-3 in cells with high levels of active Ras. By fusing half of the split protease to Ras and the other half to a domain that binds to activated Ras, they were able to reconstitute active protease in the presence of active Ras. The protease then cleaved an engineered variant of caspase-3 that is localized to the plasma membrane so that it is near the now Ras-associated protease, resulting in cell death.

**Figure 5.**
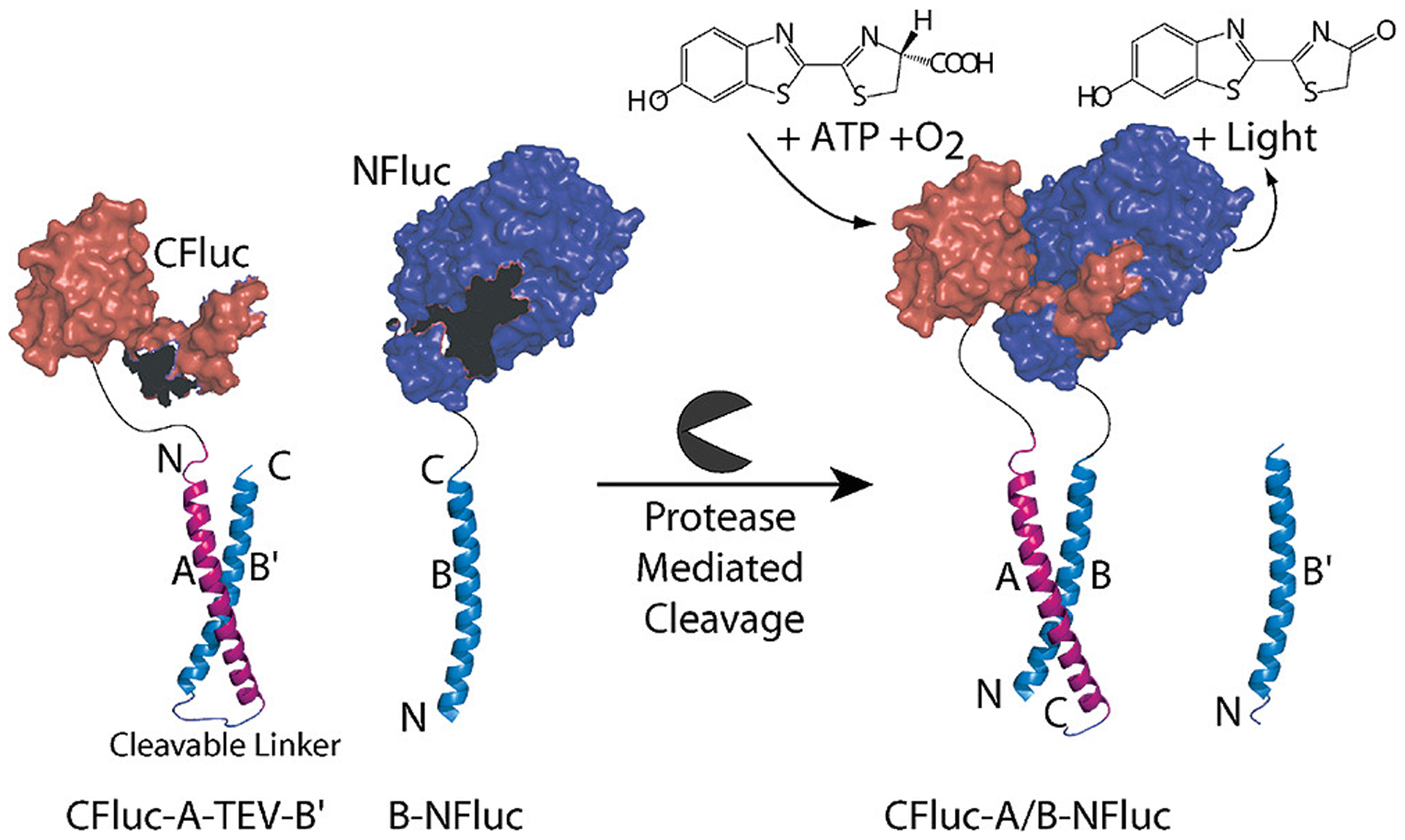
Split protein activation following protease addition. Here the firefly luciferase is separated into a C-terminal (CFluc) and N-terminal half (NFluc) both containing additional helices that can form a coiled-coil to bring both halves together to reconstitute the luciferase. This interaction can only occur when the autoinhibiting coil is released through addition of the tobacco etch virus (TEV) protease. Other reporter proteins can be similarly broken into two halves, including other proteases to generate extended logic cascades. Reprinted with permission from Shekhawat, S.S., Porter, J.R., Sriprasad A., and Ghosh, I. 2009. An autoinhibited coiled-coil design strategy for split-protein protease sensors. J. Am. Chem. Soc., 131, 15284–15290. [81]

Similar to the CHOMP system, split-protease-cleavable-orthogonal-coiled-coiled (SPOC) based logic circuits were developed, in which split proteases are reconstituted in human cells by the formation of coiled-coils regulated by orthogonal proteases. In the absence of protease, an autoinhibitory coil is covalently linked to a target coil that is linked to the protein of interest (either a reporter or half of a split protease). Adding protease to cleave off the autoinhibitory coil allows for a different coiled-coil to displace the autoinhibitory coil. When this displacing coil contains the other half of a split protease, the protease is reconstituted and can be used to cleave the linker of another coil, allowing for logic cascades. Previously, this framework was used to construct an AND gate in an in vitro translation system by using a protease to cleave between the target and autoinhibitory helices [81] (Figure 5). Fink et al. expanded on this work by using human cell lines, inducing dimerization of the coiled-coils by addition of small molecules, and allowing for negation by introducing cleavage sites between the split protease and the target coil. They constructed a 3-layer logic cascade using 3 orthogonal proteases, which in principle could be extended with the addition of more orthogonal proteases or different small molecules for inducing coiled-coil dimerization. Compared to other methods such as CHOMP (Gao 2018) and transcription-based systems [82], SPOC is much faster, producing the output within 5 minutes rather than hours.

## 3. Nucleic acid-based logic gates

### 3.1 Catalytic (entropy-driven) toehold-mediated strand displacement

**Figure 6.**
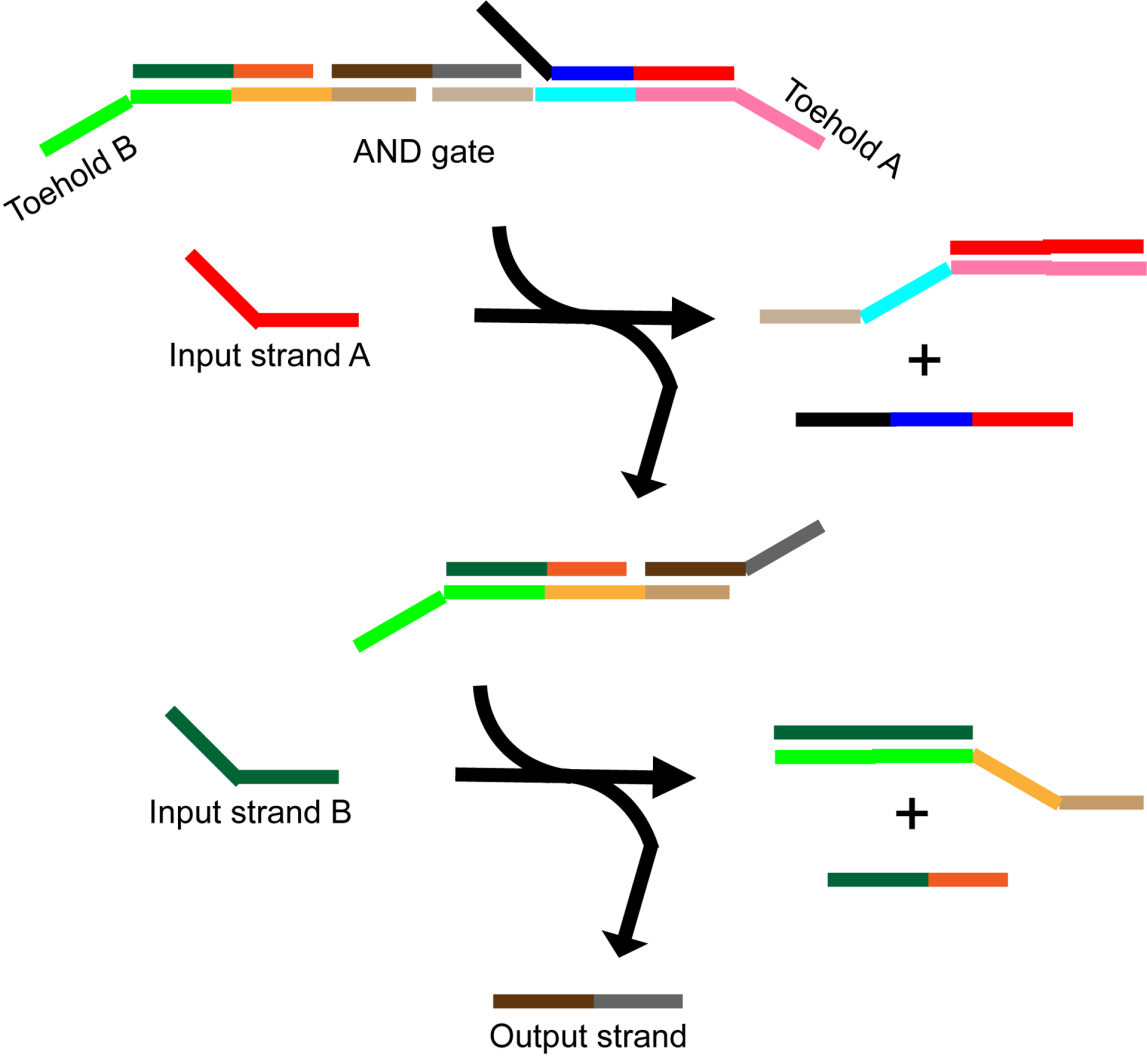
Schematic of a toehold-mediated strand displacement AND gate. Input strands Aand B displace the complementary strands from the AND gate on either side of the output strand. The output strand is released only when both input strands hybridize with the gate.

While most catalysts in biological systems are protein enzymes, nucleic acids can also catalyze reactions and undergo allosteric regulation, allowing for the construction of logic gates. A common foundation for constructing logic gates with molecules of DNA in bulk solution is toehold-mediated strand displacement. In this method, signals are carried by single-stranded DNA with two recognition domains that can hybridize with the two strands of the gate that the signal is associated with. Each gate consists of double-stranded DNA, with one strand having a short overhang called a toehold in addition to the displacement domain. Both domains are complimentary to an input strand. The input strand is single-stranded DNA that is complimentary to the toehold and the displacement domain will preferentially displace one of the strands of the gate by first hybridizing with the toehold before proceeding by branch migration. After hybridizing with the toehold, the input strand hybridizes with the displacement domain, displacing the other strand that was initially part of the double-stranded gate. The displaced strand, which contains a sequence complimentary to a different toehold than the displacing input strand then proceeds as an input for the next strand displacement reaction at the next gate in the circuit. Two-input AND gates can be created by using displacement of a strand by the first input to reveal a toehold that the second input can use to displace the output strand (Figure 6). OR gates can be created by having multiple single-input gates with the same output [83]. The outputs from strand displacement cascades can be reported by displacement of a DNA strand with a fluorophore from its complement containing a quencher [84].

**Figure 7.**
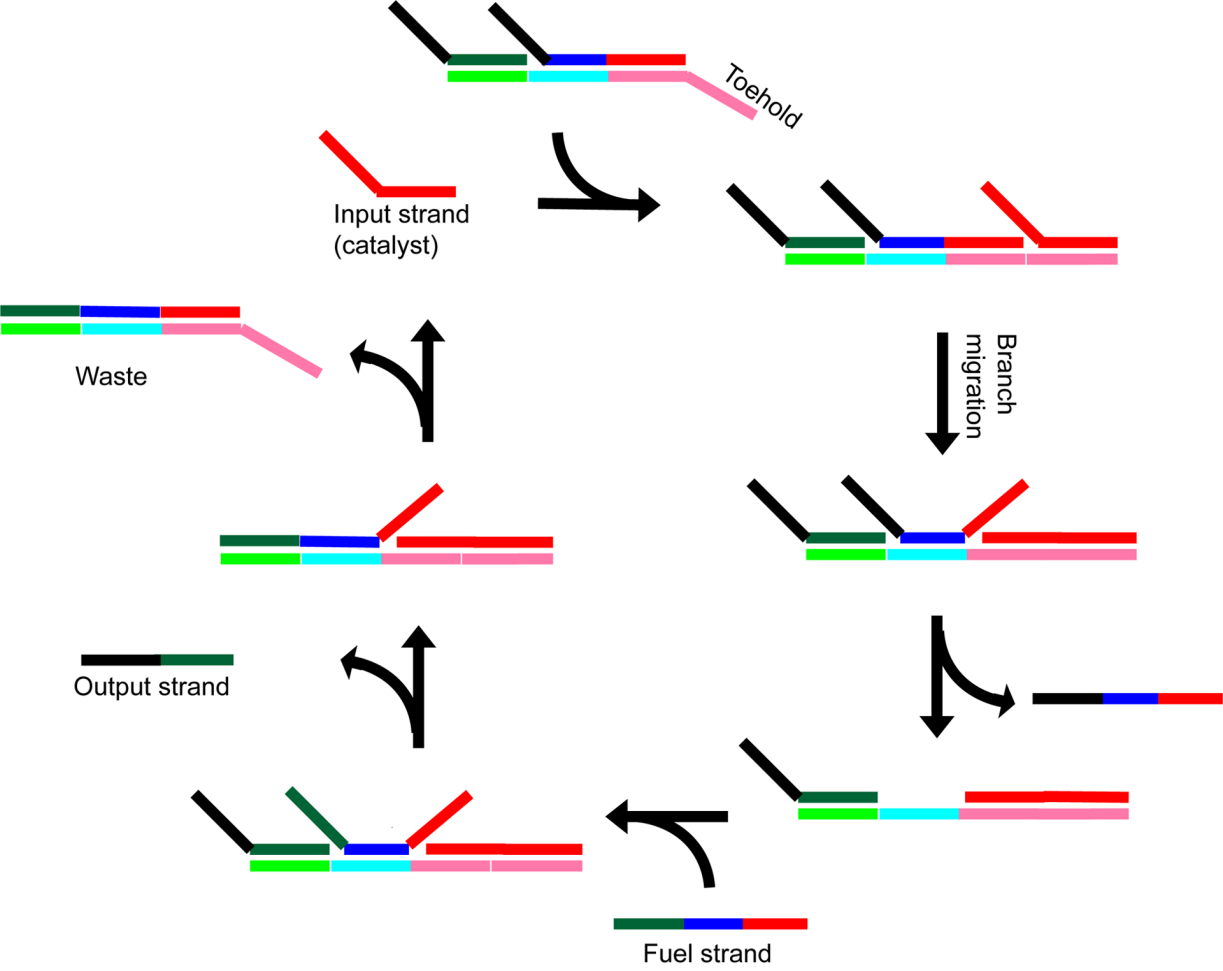
Schematic of toehold-mediated strand displacement using a seesaw gate. Different DNA sequences are shown in different colors, with black representing regions that do not form base pairs. Shades of the same color represent complementary sequences. First, the input strand hybridizes with the toehold (pink). The input strand further hybridizes to the gate via branch migration and displaces the initial complementary strand (black-blue-red). The fuel strand displaces both the output strand and input strand. The input strand can then continue the cycle with another molecule of gate DNA. The output strand can act as an input strand for a different gate.

An extension of the toehold-mediated strand displacement method is the use of toehold exchange and “Seesaw” gates (Figure 7). “Seesaw” gates rely on a “fuel” strand that allows the input strand to be regenerated at the end of each cycle so that only a low concentration of input strand is required to release all of the output strands from a gate. After the input strand hybridizes with the gate, the “fuel” strand displaces the input strand, allowing it to react with another gate and amplifying the input. Because the overall reaction for toehold exchange does not include the input strand, the input strand can be thought of as a catalyst for the displacement of the output strand by the fuel strand [85,86]. Double-stranded DNA that is complimentary to the input, called a threshold, can prevent hybridization of the input with the gate until a given concentration of input has been reached. Using thresholds and fuel together ensures that no output strands will be released (i.e. the output is 0) until a minimum number of input strands have been displaced. For any number of input strands above the threshold, the maximum number of output strands will be released (i.e. the output is 1), allowing for binary behavior. Without any thresholds or fuel, an integrating gate can be created where the output is simply the sum of any number of inputs, where each input has a sequence complementary to the integrating gate. AND and OR gates can then be constructed by placing an amplifying gate after an integrating gate and setting the threshold appropriately, with an AND gate having the threshold set above the maximum possible amount of one input. These logic gates can be chained together to form circuits of arbitrary complexity using dual-rail logic to construct complex logic functions from only AND and OR gates, with NOT gates realized by swapping the two wires of an input and output signal [87–89]. DNA logic gates can be constructed in the solution phase or with the gates immobilized on a surface [90–93] , and can even output signals on a timer [94,95]. Huang and colleagues [96] expanded the catalytic strand displacement mechanism described above to create complex logic circuits, including three-input AND-AND and AND-AND-NOT gates as well as the first DNA-only two-output Comparator and Feynman gates.

### 3.2 Using DNAzymes as part of logic gates

**Figure 8.**
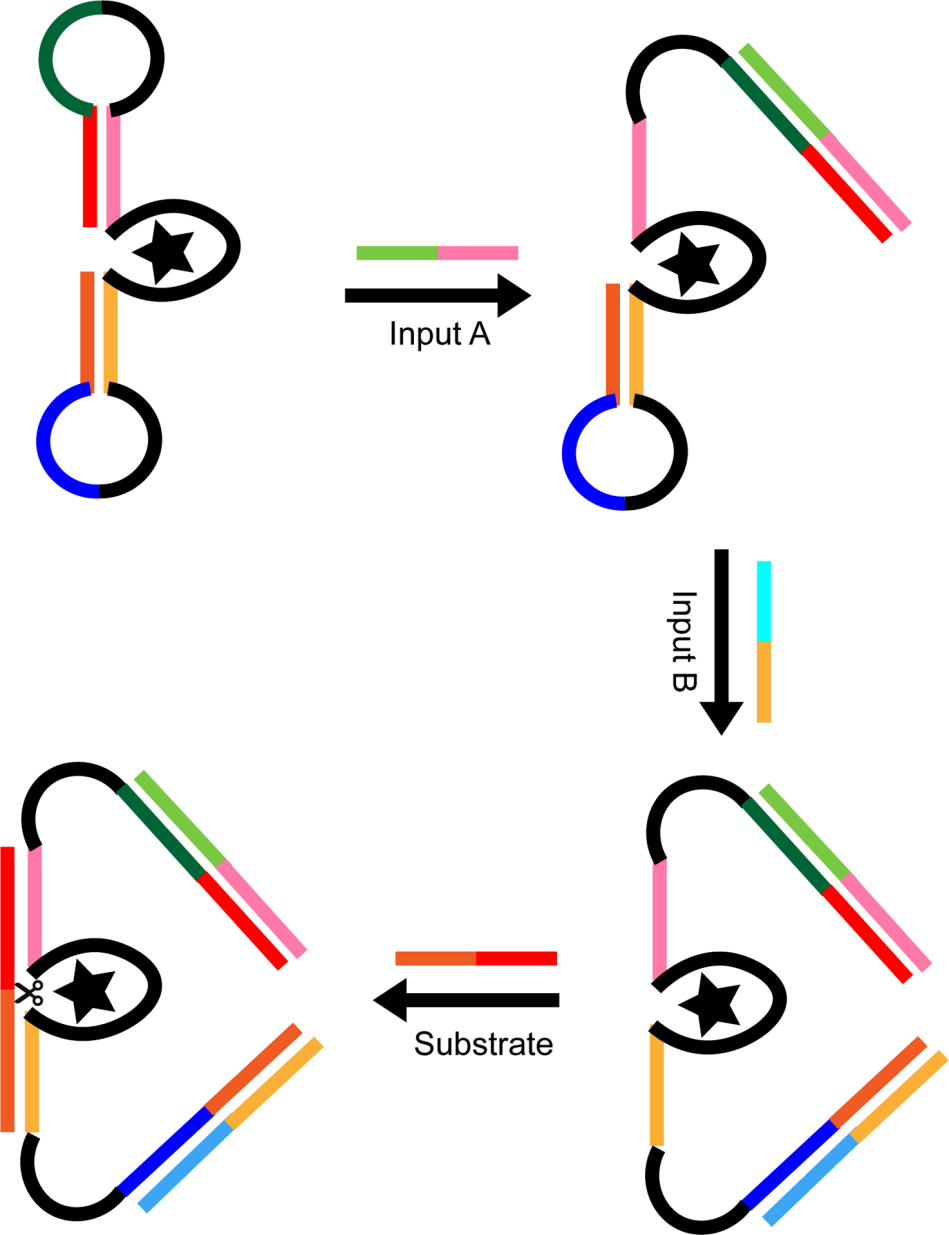
Schematic of a DNAzyme-based AND gate. Different DNA sequences are shown in different colors, with black representing regions that do not form base pairs. Shades of the same color represent complementary sequences. In the absence of input strands, the regulatory loops prevent the substrate strand from binding to the active site (represented by a star) and being cleaved. Binding of both input strands allows the substrate to bind at the active site, resulting in cleavage of the substrate strand.

Instead of the toehold-mediated strand displacement mechanism described above, logic gates can also be constructed by using strand displacement to allosterically regulate the catalytic activity of DNAzymes [97–99] (Figure 8). Early work with DNAzymes used the E6 DNAzyme, which cleaves a fluorogenic RNA substrate. The E6 DNAzyme can have its catalytic site either blocked or revealed upon binding of complimentary DNA to a regulatory stem loop, with logic gates created by using multiple stem loops around each catalytic site [83,100,101]. Multiple inputs and different fluorophores can be used, allowing for more complicated circuits [101–110]. By using allosterically regulated DNA-based ligases, the output of a logic gate can be oligonucleotides that can act as an input for another logic gate, allowing circuits to be constructed from layers of logic gates [111]. Similar systems using RNA-based inputs or catalysts and DNA substrates have also been developed [112–114], including systems that use substrate release to activate a downstream DNAzyme by strand displacement [115,116] and systems that release small molecules [117].

### 3.3 Catalytic G-quadruplexes for visualizing output of DNA logic gates

**Figure 9.**
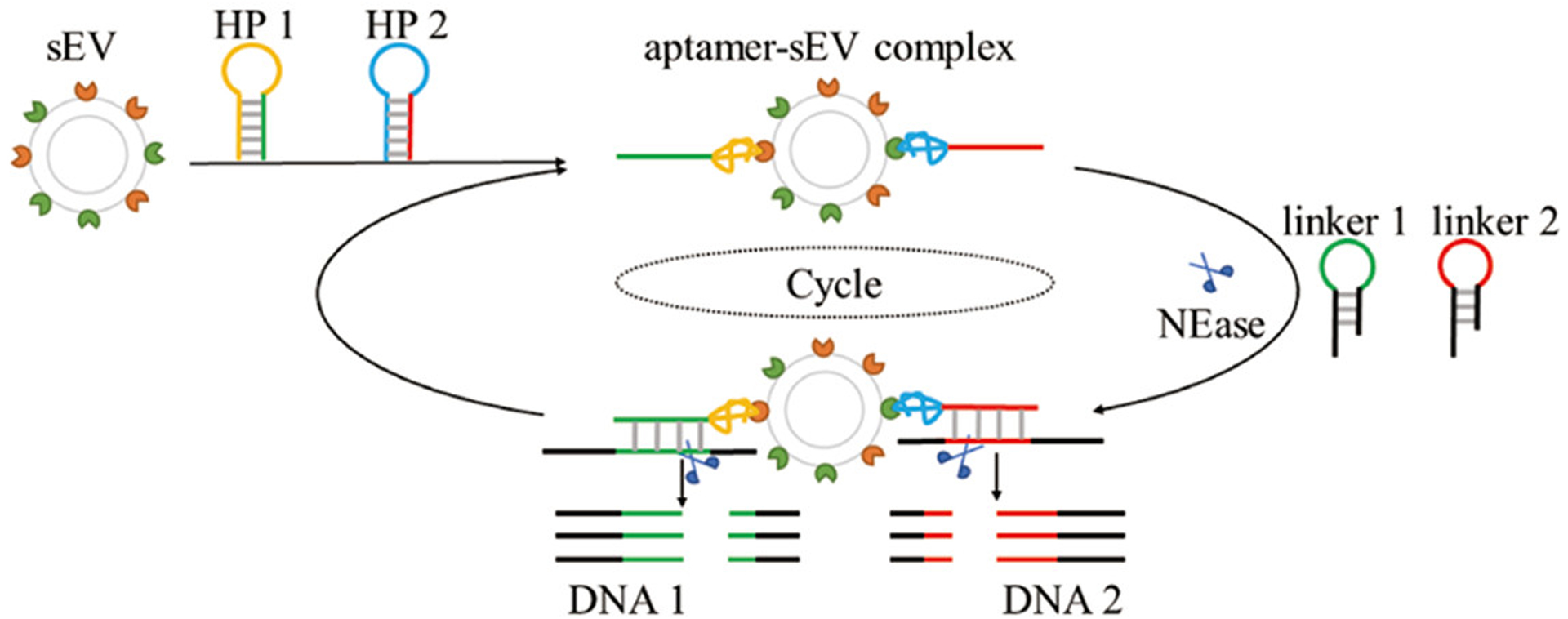
Dual aptamer assisted enzyme signal amplification. DNA hairpin structures 1 and 2 (HP1, HP2) can bind protein biomarkers on the surface of a small extracellular vesicles (sEV) leading to the release of the hybridization region (red and green parts of strands) that are complementary to regions in linkers 1 and 2 (color-coded the same). The restriction endonuclease (NEase) then recognizes and cleaves the new duplex. The resulting DNA1 and DNA2 can then have further downstream effects. Reprinted with permission from Yu, Y., Guo, Q., Jiang, W., Zhang, H., and Cai. C. 2021. Dual-aptamer-assisted AND logic gate for cyclic enzymatic signal amplification electrochemical detection of tumor-derived small extracellular vesicles. Anal. Chem., 93, 11298–11304.

G-quadruplexes, stacks of four planar guanine bases associated by Hoogsteen base pairing stabilized by a metal ion that are found in G-rich sequences of nucleic acids, have also been used as the basis for DNA-based logic gates. There is a large body of work on using G-quadruplexes that respond to input DNA oligonucleotides [110,118–121] or chemical conditions [122,123] by detecting the G-quadruplex via fluorescence, but our focus in this review is on the use of catalytically active G-quadruplexes that form in response to aptamer binding as part of logic gates. In these systems, a G-quadruplex intercalates hemin to catalyze the reduction of H_2_O_2_, oxidizing a substrate to produce a colored product or chemiluminescence analogous to horseradish peroxidase [124]. These catalytic G-quadruplexes have been used for the detection of metal ions [125–127], small molecules [128], and specific single-stranded DNA sequences. Catalytic G-quadruplexes are particularly well-suited to determine the output of a DNA logic circuit because the output can be detected visually without the need for potentially expensive equipment and G-quadruplexes can be formed in multiple ways. G-quadruplexes can be released from a duplex or triplex by strand displacement [129] or formed from two separate strands in response to strand displacement [110,118] or input oligonucleotides [130]. A notable example of a DNA-based logic gate with for detection can be found in [131] (Figure 9). Yu et al. used DNA aptamers that recognize protein biomarkers at the surface of extracellular vesicles. The aptamers had an exposed toehold for hybridization with another strand of DNA upon binding the proteins. After hybridizing, DNA is cleaved by an endonuclease, releasing the DNA and allowing for signal amplification. One of the released DNA strands hybridized with DNA conjugated to a gold electrode and the other hybridized with the first strand and formed a G-quadruplex with peroxidase activity, resulting in an electrical signal only when both protein analytes were present.

## 4. Discussion

The logic gates presented here that depend on protein-level regulation can be compared against those that regulate at the level of transcription. In general, logic gates that depend on protein-level regulation are faster than transcription-based logic gates, occurring on the timescale of minutes rather than hours to days [46,132]. This is a major advantage because biological processes such as glucose metabolism occur too quickly for efficient regulation at the transcriptional level [132]. However, an advantage of transcription-based approaches is that when the final output of the logic operations is regulation of transcription, a wider range of outputs are available. For example, in the cellular logic circuits reviewed in this article, the outputs of the circuits were limited to fluorescence, activation of a specific kinase that could be engineered to respond to light, and cleavage of an engineered caspase. In contrast, transcription-based approaches can use proteases to release transcription factors for virtually any gene [44,133].

Logic gates that rely on the detection of specific molecules, such as those presented in Section 2.3, will not be as useful for performing logic operations in cells because they are not based on generalizable principles and would experience interference from cellular metabolites. The GHD-CaM fusion system presented in Section 2.2 however, can potentially be very useful for biosensing applications because it can be used with antibodies for detection of virtually any protein of interest. Similarly, the protease-based methods described in Section 2.4 are able to be modified to perform logic operators on a variety of protein targets. Detection of protein can in principle be coupled with drug release, although coupling the output of the logic gates with drug release in the manner described in Section 2.3 would be challenging to achieve in a biological system.

Some of the logic circuits in this review ultimately release molecules from hydrogels by production of H_2_O_2_. These logic circuits could be combined with H_2_O_2_-releasing hydrogels that have been developed for therapeutic applications such as wound healing [134,135]. Currently, the H_2_O_2_-releasing hydrogels release H_2_O_2_ from the oxidation of glucose catalyzed by glucose oxidase. By using protein-based logic gates to regulate glucose oxidase activity, such as in the work described in Section 2.3, the formation and release of hydrogels can be regulated by logic gates. Similarly, catalytic G-quadruplexes that catalyze the production of H_2_O_2_ could be used as alternatives to glucose oxidase in these systems.

DNA-based logic gates have several advantages over protein enzyme-based logic gates for in vitro computations. One advantage is that they are easier to scale up due to the relative simplicity of Watson-Crick base pairing, as complimentary DNA sequences with well-understood displacement kinetics can be designed and synthesized much more easily than allosterically regulated proteins or protein-protein interaction interfaces [136]. An impressive example is reported in Cherry et al., where a DNA strand displacement-based logic circuit was used to distinguish between two handwritten numbers on a 10×10 pixel grid using 104 different DNA molecules [137]. A major disadvantage of using DNA for molecular logic circuits over proteins is speed – the image recognition circuit took hours to produce a result, on a similar timescale to transcription-based logic. Another disadvantage, not unique to DNA, is that scaling up to more complex logic circuits is challenging due to the large numbers of unique molecules needed and the extra time required to carry out the calculations.

While there are many examples of protein-based enzymatic logic gates that have been implemented in living cells [66,79,80,132], applications for DNA-based circuits integrated into cells have been more limited. DNAzymes have been introduced into cells for gene silencing and biosensing [138,139]. One of the major limitations is that DNA introduced into cells is susceptible to cleavage by nucleases. One strategy for improving the stability of DNAzymes, aptamers and so on is to evolve the sequence with desired activity in biological fluids [138], but this is not feasible for complex DNA logic circuits where the sequences must be carefully designed. There are many recent examples of DNA-based biosensors where fluorescence outputs are used to detect multiple types of molecules at the same time, including RNA and proteins at the cell surface [140–146]. Because the output is fluorescence, these biosensors have not been used for downstream logic processing. Chang et al. designed a DNA aptamer-based biosensor that forms a toehold when both analytes are present at the cell surface, allowing for downstream biomolecular logic processing [147]. Systems that use DNA logic circuits for delivery of therapeutics have been proposed or supported by in vitro experiments, but have not yet been implemented in cells. For example, a molecular device based on DNAzymes allosterically regulated by glucose has been proposed for management of diabetes [148], but such a device has not been experimentally realized. In another example, an RNA-cleaving DNAzyme that can cleave RNA to prevent transcription of cancer-associated mRNA in response to high concentrations of cancer marker RNA has been demonstrated to work in vitro [149] but not yet in living cells.

Biomolecular logic gates are likely most useful for diagnostics, while therapeutic applications are more challenging. For DNA-based biosensors, a logic circuit with inputs based on aptamer binding and an electronic output that relies on G-quadruplex formation as the output like that used by Yu et al. [131] is the most promising for diagnostics based on cell surface markers. While logic gates are not strictly necessary for detection of multiple analytes, as different fluorescent signals can be used, DNA logic circuits can be useful for simplifying the output when different combinations of analytes are detected. We think the most promising protein-based logic gate biosensors are those that can be conjugated to antibodies for detection of different proteins, such as the GDH-CaM system from Guo et al. described in Section 2.2 [61]. For protein enzyme-therapeutics where a rapid response to changing conditions is required, the protease-based logic circuits that rely on degradation or activation of protease are likely to be the most useful of the systems discussed in this article, as they have already been demonstrated to work inside cells.

## 5. Conclusions

The logic gate framework can be used to conceptualize, and in some cases simplify, complex regulatory networks in biology. In metabolic engineering, this framework can then be used to optimize production of high value chemicals [1,2]. Advancements in biomolecular engineering will provide additional protein- and nucleic acid-based logic gates [43,150]. Protein- and nucleic acid-based logic gates can then be programmed to interact with each other [151], and catalyst-based logic gates can similarly interact with non-catalyst-based logic gates (e.g. transcription factors [152,153]). These logic gates have the potential to integrate multiple signals and thus make intelligent decisions in therapeutic and diagnostic applications. Several applications have already been realized, including in the detection and treatment of cancer (e.g. [140]) and the detection of various metabolic diseases (e.g. [154]).

## Figures and Tables

**Table 1.** Fundamental Boolean logic gates.

Input Values		Output Values							
A	B	AND	OR	INH	XOR	NAND	NOR	IMP	XNOR
0	0	0	0	0	0	1	1	1	1
0	1	0	1	0	1	1	0	1	0
1	0	0	1	1	1	1	0	0	0
1	1	1	1	0	0	0	0	1	1
